# The Promotion of Osseous Development

**Published:** 1884-08

**Authors:** George Watt


### COMMUNICATIONS.

### The Promotion of Osseous Development.

BY GEORGE WATT.

Read before the Mad River Valley Society.

In the statement of the proposition it is taken for granted that bone may be developed, and that such development may be encouraged or promoted.

In a constitution perfectly healthy and well balanced, the bony tissues are developed exactly to the extent that they are needed; but in this proposition it seems to be taken for granted, also, that the process of development is not always well balanced. It may be easy for one constitution to develop bone, while it partially fails in the development of muscular tissues. In another, the constitutional tendency may be toward the development of nervous tissue, while another runs to fibrous tissue in general.

The question now comes up: Can we, in cases of badly balanced development, do anything to restore or to establish a constitutional equilibrium? All will admit that by violation of the laws of health we can do much to bring about an unbalanced state of the constitution; and it seems but reasonable that we ought to be able to do some good by a reversal of the process.

In a "one-horse shay," and in any machine, the weakest place must stand the strain, and the way to guard against its breaking, in order that it may be honestly worn out, is to "make that place as strong as the rest." We may thus get a hint on the subject before us. If an organ or function be weakened, so that it partially fails to do its part in the economy of the system, one of two things seems to be called for. We may endeavor to render its work easy, so that it can perform it, notwithstanding its debility, or we may try to arouse it to increased energy of action. Either process may, for the time, restore or establish the desired equilibrium; but the latter mode may be similar to the plan of whipping or spurring the exhausted horse. Just as in case of the horse, we must inquire whether the defect results from sluggishness or from exhaustion. If from the former, we may stimulate, but if from the latter, we would only do mischief by such a course. We saw this plan illustrated when a boy. A shiftless man, with a team, stopped to begged a dinner for himself, and some thread to make a whip-cracker. While eating and repairing his whip, his horses stood tightly reined in the hot sunshine, and we were not surprised to see that he was fatter than his horses.

All tissues are formed from the blood. "For the life of the flesh is in the blood" is a physiological truth, taught by infallible authority, many long centuries before Hippocrates. Hence whatever is to be built into the body must be passed to it through the blood. And so it follows that no tissue can be nourished unless the blood contains the materials necessary to its nutrition—the materials of which it is composed. It does not follow, however, that an organ will certainly be nourished because the blood contains the desired nutritive principles, for it may not be able to appropriate that which is prepared and ready for it, just as a man may starve in full sight of food, because not able to take it.

It must be always borne in mind that the nutritive functions create nothing. They cannot use that which is not already formed. And let it be remembered, too, that all organic bodies are built up in strict accordance with the laws of chemical combination. Vitality modifies chemical action, but never contradicts or destroys it. It may determine what combinations can take place under certain circumstances, and what can not. It may prevent one, and promote another combination; but, in order to promote any, the materials must be there to combine.

Furnishing food for vegetable life is commonly called manuring; and when feeding his plants the intelligent farmer tries to know what his soil contains, and what are the constituent materials of his plants, and if the soil is short of these constituents he supplies them. An instructive case of this kind is recalled by memory. A minister of the gospel owned a farm. He called on a man who had some reputation as a chemist, and asked him if he was prepared to analyze soils, and was told that wan't of time was the only lack, but that would prevent his giving any attention to the soil; but the chemist added: "If you tell me what is the trouble, I may help you without an analysis." "Well," said he, "it will not produce anything." "But that is a slander on your field," said the chemist; "any field you have will bring something. Can you raise straw, or corn-stalks on it?" "Why, yes," said the man, "great crops, but no grain." Knowing the land to have a sufficiency of lime and magnesia, the chemist told him to give the field a dose of phosphorus, otherwise bone ashes. The remedy was tried, and a wheat crop of twenty-five bushels to the acre, followed by a crop of corn of over seventy, was the result.

The same principle governs in animal life. Just as the rootlets of the plant cannot find and use from the soil that which is not there, so a tissue cannot appropriate from the blood ingredients not contained in it. We have already alluded to the fact that chemical laws are not violated in the formation of living growths; and as quantity of matter is one of the modifying circumstances of affinity, it follows that when anything essential to the development of any texture is deficient in quantity in the blood, the development of that texture is promoted by furnishing it in greater proportions to the circulating fluid.

In ordinary constitutions, some essential constituents are more readily assimilated than others; and this is not more a matter of surprise than is the fact that some foods are digested with greater facility than others.

Bone, like other organic matter, is composed mainly of carbon, hydrogen, oxygen, and nitrogen. These constitute its diet, but not its desert. For this it wants salts, and similar materials; and an important, if not the most important one, is the subphosphate of lime. At the risk of disturbing a friend, we will state this is not the neutral phosphate, but a much more stable salt—one that will endure a white heat, without decomposition. To distinguish it, some of the older chemists have recommended that it be called simply "bone phosphate." Not only is this ordinarily the most important salt concerned in osseous development, but it is often, if not always, the most difficult to obtain and assimilate. And this statement is all the more confirmed by the fashionable habit of separating the greater portion of the phosphate from the food producing grains before using them. As early as 1854, we called earnest attention to this, recommending that whole grain flour be substituted for the white family flour of commerce. We had been administering the bone phosphate as a medicine, to aid in bony development, from the time we began practice, in 1844, and we never had cause for discouragement in its use. Good results were as uniformly obtained from its administration as from other medicines. Prof. Taft will remember a case thus treated, in 1849 and 1850, with the most satisfactory results. At the risk of hearing something told you before, please listen to a report of this case:

Mrs. M., aged 25, was the mother of two children. She had suffered much from defective dentition, and needed an upper artificial denture. The two children had scarcely any sound teeth, and were often crying with toothache. A third conception had taken place. From about the third month of utero-gestation, at our suggestion, she used daily, and usually three times a day, the bone phosphate. From a pale, emaciated woman she became full-faced and rosy-cheeked; had an easy, natural labor; the babe weighing over twelve pounds; and this child, in time, had a most excellent set of temporary, followed by an equally good set of permanent teeth. The phosphate was continued during the period of lactation. The contrast between this child and the other two was too decided to be accidental; and the case is merely a type of others treated in a similar way.

In 1855, we mentioned this case to Dr. Elisha Townsend and a few other dentists. One of them reported it to his family physician, and requested that his wife be allowed to follow the same course. The physician feared that it might render the labor more difficult, but the phosphate was administered in rather limited quantities. The result was quite satisfactory, and continued to be so till all but the third molars were developed. We then lost sight of the case. The other children in the family had very defective teeth as compared with the one aided by the bone phosphate.

The phosphate used in these cases was prepared by dissolving bones in hydrochloric acid, precipitating with ammonia, and washing and drying the precipitate. We do not think any *better* form of the medicine has been suggested, but some have been prepared so as to be more pleasant and palatable.

But food is better than medicine. Let foods rich in the phosphate be used. The lean meat of beef, mutton, fowls, etc., answer a good purpose. Cracked wheat, oatmeal grits, if properly prepared, are also valuable. From experiments tried long ago, we were led to believe that veal and pork are not so rich in the phosphate as are the meats above mentioned.

Some authors speak of the subphosphate being unreliable, if not inert, as a medicine, on account of its insolubility; but their objection loses force through their accompanying statement that it is *highly soluble* in hydrochloric, acetic, and lactic acids. We may add that it is also highly soluble in solutions of carbonic acid; this being the agent that holds it in solution in normal saliva, which can be well remembered by association with the fact that it is the removal of the free carbonic acid from the saliva, by ammonia, that causes the precipitation of this salt in the form of tartar. As all four of these acids are common in the stomach, there can be but little difficutly in obtaining a solution of the salt in it during the process of digestion.

Others have objected to the use of this salt on the principle that inorganic matter cannot be assimilated by the animal economy. But as this drug is obtained by solution and precipitation from *bones*, the inorganic objection falls.

If readily obtainable, it is well to use some of the more elegant preparations, such as wheaten phosphates, lacto-phosphate syrup, etc., but sometimes a fatal delay occurs, because these are not at hand. If not already prepared, the preparation of the salt is so simple, and the chemicals needed in the process are so readily obtainable, that there is no excuse for delay or negligence.

In considering this topic, it is to be understood that all the nutritive functions are to be in good working order. Sometimes the digestive apparatus and the assimilating powers are so feeble that they are scarcely able to develop any of the tissues. If possible, these are to be restored to health and strength preparatory to special development; for we can scarcely promote osseous development when the vital powers are so enfeebled. And this state of affairs suggests counsel and co-operation with the family physician, provided he is a gentleman, and not an empiric.

To sum up, let us bear in mind that all tissues are built up from materials taken from the blood; that the nutritive functions create nothing, but only digest, assimilate, and appropriate; that, therefore, unless the needed materials are in the blood, in the required quantities, there must be defective tissue building, or no develpment of tissue at all; that when the needed materials are abundant, there must be, other things being equal, a better and fuller development than when they are deficient in quantity.

We have tried, in a hurried way, to discuss the general principle, rather than to give recipes. Such a course is more likely to call out original thought.

